# Registered nurses and what may constitute their leadership in the home healthcare context

**DOI:** 10.1186/s12912-025-03171-0

**Published:** 2025-05-26

**Authors:** Malene Søiland, Trude Furunes, Margareth Kristoffersen

**Affiliations:** 1 Department of Caring and Ethics, Faculty of Health Sciences, University of Stavanger (UiS), PO Box 8600, Forus, Stavanger, 4036 Norway; 2 NHS–Department of Leadership and Service Innovation, Faculty of Social Sciences, University of Stavanger (UiS), PO Box 8600, Forus, Stavanger, 4036 Norway

**Keywords:** Home healthcare, Individual interview, Leadership, Nursing activities, Registered nurses

## Abstract

**Background:**

Registered nurses who are not employed in formal leadership positions are assumed to practice leadership, yet there is limited knowledge of what this entails. This study aimed to identify what may be recognised as critical aspects constituting leadership in registered nurses’ daily interactions in the home healthcare context.

**Methods:**

A qualitative study design with a hermeneutical approach was employed. Data were collected through individual interviews with registered nurses working in three home healthcare contexts in three Norwegian municipalities. Data were analysed using thematic analysis.

**Results:**

The results are presented as three critical aspects that seem to be recognised as constituting leadership in the home healthcare context: (1) Negotiating accountability for nursing activities, (2) Gaining an overview of nursing activities, and (3) Managing nursing activities within a limited time.

**Conclusions:**

In this study, leadership appears to emerge when the registered nurses act as meaning-makers in daily interactions with their colleagues. The nurses act as meaning-makers to achieve a specific goal: to accomplish nursing activities to care for patients needing help. Acting as meaning-makers involves the nurses being aware of and actively recognising negotiating accountability for nursing activities, gaining an overview of nursing activities, and managing nursing activities within a limited time. These are critical aspects that may constitute leadership in their daily interactions.

## Background

Over the years, the home healthcare context in Norway [1], as well as globally [2], has changed due to demographic shifts. People are living longer with complex chronic conditions [3] and there is a focus on early discharge from hospital to primary healthcare [4], with services being offered in home healthcare [5]. In Norway, this re-organization of healthcare has resulted in high demands on home healthcare nurses [6, 7], who are registered nurses expected to have responsibility for sicker patients in all age groups [8, 9]. Within the domain of nursing, nurses are accountable for patients’ care, and this requires them to utilize professional knowledge for their nursing judgments and apply evidence-based decision-making to provide high-quality patient outcomes [10]. Hildegaard Peplau, who is an American nursing theorist [11], describes professional nursing as an interpersonal process because it involves interaction between two or more individuals with a common goal. The nurse is trained to recognise and respond to the needs of patients, and leadership is embedded in taking an active role in nursing care. Peplau [11] explains that nurses demonstrate leadership by involving everyone participating in nursing care, sharing the work tasks, making decisions collectively, and planning nursing activities through open discussions. Other researchers have also underlined that nurses practice leadership [12, 13]. The nurses’ responsibility is often described as an individual ability to provide direction with a common goal, influence nursing care and empower others [13, 14]. This implies that nurses’ leadership involves interacting with other health professionals and patients on both organizational and interpersonal levels [15], including coordinating nursing activities that align with the patient’s needs and organizing unforeseen work tasks [16]. More concretely, their interactions include considering the nursing competence required to support patients living at home [5, 17], delegating nursing activities, such as wound care, to their colleagues [18], guiding them to perform good nursing care on their own [19–21], evaluating colleagues’ competence to ensure a safe and supportive work environment [15] and following up to ensure colleagues have managed their nursing activities [18]. In a Swedish quantitative study of nurses working in the context of home healthcare, the nurses perceived challenges in their practice of leadership [22]. They struggle to maintain a balance between the resources available and the demands placed on them, such as having to make decisions and perform accurate assessments, including choosing the right interventions and managing patients’ acute symptoms. Additionally, colleagues sometimes impose demands on nurses to act, particularly when patients’ conditions change [22]. A constant lack of time often makes it hard for nurses to keep up with all the nursing activities [16, 23], especially when they also have to organize unforeseen work tasks [16]. Even though nurses consistently strive to manage their time and provide care, a lack of time may compromise the quality of nursing care [5]. Nurses raise concerns about their responsibility to maintain the quality of nursing care because they often also have to evaluate their colleagues’ competence to ensure a safe and supportive work environment [15]. Research also reports that leadership in a home healthcare context includes organizing unforeseen work tasks, often requiring nurses’ knowledge of the organization and collaboration, as well as coordination of nursing activities that align with the patient’s needs [16].

Although the amount of literature on nurses’ leadership has significantly increased in recent years, research mainly focuses on hospitals and nursing homes [14]. Several recent studies have discussed the need for more research on registered nurses’ practice of leadership within the home healthcare context [15, 24, 25]. There are several definitions of leadership [26], and the concept is often used interchangeably with management [27]. While these two concepts may overlap [28], they are not synonymous [27]. Management can be described as planning, budgeting, and resource allocation to achieve organizational goals [29]. Leadership, on the other hand, can be understood as a relational practice, and thus this study is framed by the concept of leadership as described by the American professor Joseph A. Raelin [30, 31]. Raelin [30] explains that, as a relational practice, leadership occurs through a coordinated and collaborative effort among the participants in an activity where the purpose is to achieve a goal. More specifically, leadership concerns meaning-making [31]. Within a group or organization, meaning-making involves articulating what the participants are trying to achieve together. Raelin [31] states that meaning-making expresses a collective sense of what the group stands for and portrays an image of what the group is doing or not doing, identifying what is missing or not happening. Anyone in a group can contribute to meaning-making processes. However, a meaning-maker is usually an active listener and an expressive user of techniques to articulate meaning. A meaning-maker can use humour to describe a situation, synthesize the facts, look for patterns in situations, or turn a problem upside down and consider it from a new perspective. Thus, in regard to practising leadership in the home healthcare context, this study focuses on registered nurses employed in front-line positions – not in formal leadership positions – in healthcare organizations. Additionally, it focuses on what aspects of leadership may be embedded in nurses’ daily interactions, and consequently there for the taking, but require identification to be visible. Such aspects are considered critical, as they refer to what is essential to constitute leadership and what nurses recognise as constituents of leadership. In turn, acknowledging what constitutes leadership can clarify for the nurses themselves what the practice of leadership is, and facilitate their meaning-making. This study aimed to identify what may be recognised as critical aspects constituting leadership in registered nurses’ daily interactions in the home healthcare context.

## Method

### Setting

The setting for this study was three home healthcare contexts representing two rural and one urban area. They all carry out nursing to home residents, regardless of their age, diagnosis, or disability status. In Norway, home healthcare is a municipal responsibility, and all people living in or visiting the municipality have a legal right to receive home healthcare if they are in need of help. In the home healthcare context, nursing care is provided by nurses and colleagues with varying levels of education. In addition to colleagues who have graduated with a bachelor’s degree in nursing, nurses commonly interact with colleagues who have less formal education, e.g. skilled health workers with an upper secondary school level health education and assistants who have not graduated in health care [1].

### Research design

The study, which is part of a larger qualitative research project entitled “Registered nurses’ practice of leadership in the home healthcare context” explores nurses’ leadership in their daily interactions in three Norwegian municipalities’ home healthcare contexts. The current study employed a qualitative design [32] with a research approach based on thematic analysis [33]. The approach was chosen for its adaptability in identifying aspects that may be recognised as critical aspects constituting leadership in nurses’ daily interactions in the home healthcare context. An inductive analysis was chosen, implying the analysis was data-driven and that there was no pre-existing coding frame for the point of departure [34]. Moreover, thematic analysis was considered beneficial in order to go beyond the manifest content of the empirical material and interpret the underlying meanings of the text, which involves examination and theorisation of manifest content [34]. Individual interviews were chosen to collect data as they allow participants to talk freely and open up for dialogue [33] about their practice of leadership. The analysis process followed a circular pattern across the interview data before landing on the exact theme through repeated reading, reviewing and refining of themes [35]. The Standards for Reporting Qualitative Research (COREQ) have been utilized in designing the study and presenting the results [36].

### Sample and participants

A purposive sample procedure [32] was used to recruit the participants. The inclusion criteria were (a) nurses with a bachelor’s degree working both day and evening shifts within home healthcare contexts, and (b) work experience amounting to approximately full-time, or in full-time positions on permanent contracts (75-100% position). Nurses in hierarchical, formal leadership positions or possessing a leadership grade were excluded. To recruit the participants, the first author approached the head managers of primary healthcare services in three municipalities in western Norway to distribute oral and written information about the study. With the head managers’ help, the first author contacted the formal leader of one home healthcare district in each of the three municipalities. Recruitment involved the first author attending meetings in each home healthcare district to orally inform all the employed registered nurses about the study. In a verbal introduction to the participants, the researcher identified herself as a researcher and a registered nurse. At these meetings, written information including details about the study aim, data collection, data management plan, and consent form was also handed out. Those who consented to participate were asked to contact the researcher by email or telephone and an appointment was scheduled. Subsequently, five, six or seven participants were recruited from each of the three home healthcare contexts respectively. In total, 15 participants were eligible. They were all female registered nurses aged 31–60 years (median 45 years) with between 2 and 30 years’ work experience as a registered nurse (median 16 years).

### Data collection

The first author (MS) collected data from January–June 2021, conducting one individual interview with each participant in an undisturbed room at their workplace. An interview guide was used to direct the conversation as to what critical aspects may constitute leadership within registered nurses’ daily interactions in home healthcare. The participants were asked to talk freely and answer an open-ended question: ‘What is nurses’ leadership within daily interactions?’ Follow-up questions were posed to elicit more nuanced information about leadership in the home healthcare context: ‘What is leadership in nurses’ daily interactions within the home healthcare context? ‘What influences leadership?’, ‘What facilitates leadership?’, and ‘What are barriers?’. During the interview, more specific follow-up questions were also asked, based on the participants’ expressions and responses: ‘Can you say more about what you are doing in your leadership in daily interactions?’ and ‘If you were to be more precise about your practice of leadership in daily interactions, what would you say?’. The interviews lasted between 45 and 90 minutes. All interviews were audio-recorded and transcribed by the first author.

### Ethical considerations

The study has been assessed by the Norwegian Agency for Shared Services in Education and Research (SIKT) (571510). In accordance with the Declaration of Helsinki [37], informed written consent was obtained from each participant before their participation. The participants were notified they could withdraw their consent voluntarily at any time. Confidentiality was guaranteed by removing all information identifying the individual participants and by using codes ranging from 1 to 15.

### Data analysis

The first author (MS) started by listening to the recorded interviews to become familiar with the content. Afterwards, each interview was manually transcribed (in total 213 pages**)** and the transcribed text was read and re-read with an open mind. While reading, the researcher made notes in the margin regarding any thoughts, ideas or questions of interest in the text related to what may be recognised as critical aspects constituting leadership. Through this familiarization, the coding process began with an initial coding of the interview text [34]. This approach started with each interview before analysing across the entire set of interview texts. When the first author found interesting transcripts in one interview, the reading moved back and forth between interviews to further identify transcripts of interest. The initial codes, such as ‘respect for leaders’, ‘deal with different personalities’ and ‘systematising activities’ were reviewed and organized, implying the authors shared their impressions and refined them into a connected code that captured commonalities. These codes were refined into initial themes, which were then identified as patterns of shared meanings [35], such as ‘lack of role clarification’ and ‘allow themselves to be a leader’. The initial themes were then reviewed again and themes with similar content were defined, collated, and named [34]. The analysis evolved as the study results were being written (see Table 1). All authors participated in the analysis process and considered whether the interpretations were valid in terms of what the text said.

**Table 1:** Examples of the analysis process

**Transcripts**	It must be stated somewhere what nursing activities I can decide about in my daily work; I want us to decide what nurses are accountable for, and what we can be allowed to decide. This is because I don’t want to make mistakes (Nurse 16). It’s when I get the feeling, or someone says to me that ‘you’re doing a good job, you performed the task in a good way’, then, I can take accountability. In other words, I notice that my colleagues and the formal leader trust me or they tell me that I’m doing my job satisfactorily (Nurse 9).
**Codes**	Reaching agreements among colleagues, implying navigating daily interactions to clarify nurses’ accountability for nursing activities. To constitute leadership as a part of their accountability, the nurses needed to clarify what is allowed to decide. Formal leaders and colleagues should affirm nurses’ accountability for nursing activities, what they are allowed to decide, and when they successfully practised activities, otherwise they will struggle with uncertainty
**Theme**	Negotiating accountability for nursing activities
**Transcripts**	When you have leadership in nursing you have an overview of everything and everything that is happening, like, how many patients need help, often it’s about twenty patients, and you have a responsibility for them even if you haven’t visited them in their homes. You have to be informed about their situation and what’s changed, or whether they have special needs to follow up (…) However, sometimes I have an overview or take the lead more, and sometimes I can relax a little (Nurse 8). We have overall responsibility for the patients and make sure that they receive the treatment they should have. We must organize the day and take control of everything we’re going to do (Nurse 5).
**Codes**	An overview involved a focus on current nursing activities, including new information and follow-up tasks for the day, the patient’s condition and whether there was any deterioration to act upon. The nurses were required to gain an overview of the performed nursing care regardless of whom had been on a home visit.
**Theme**	Gaining an overview of nursing activities
**Transcripts**	We only have the time we have, so we just have to do it as efficiently as possible, implying that we have done all the tasks of the day and properly done the tasks. Now I have started to explain how colleagues should do things themselves. When colleagues say they can’t do a task, I tell them that they must find out themselves or I can show them straight away instead of us sitting and talking about the task (Nurse 7).
**Codes**	Limited time causes the nurses to estimate how much time to spend on each nursing activity to perform activities properly. The nurses told their colleagues they had to figure out for themselves how to perform tasks, or the nurses chose to explain or show their colleagues how to perform a task.
**Theme**	Managing nursing activities within a limited time

## Results

The analysis identified three critical aspects that seem to be recognised as constituting leadership in registered nurses’ daily interactions in the home healthcare contexts: (1) Negotiating accountability for nursing activities, (2) Gaining an overview of nursing activities, and (3) Managing nursing activities within a limited time. These aspects are interrelated and seen as integrated into nurses’ daily interactions.

### Negotiating accountability for nursing activities

Negotiating accountability for nursing activities refers to a process in nurses’ daily interactions that can be seen as one aspect that may constitute nurses’ leadership in the home healthcare context. Such an aspect is critical, as it involves reaching agreement among colleagues, implying navigating daily interactions to clarify each nurse’s or colleague’s accountability for nursing activities.

*What nursing activities I can decide about in my daily work must be stated somewhere; I want us to decide what the nurses are accountable for*,* and what we can be allowed to decide. This is because I don’t want to make mistakes* (Nurse 16).

As the nurses themselves were not familiar with how to comprehend their accountability for nursing activities, i.e. what they were allowed to decide, their leadership needed to be affirmed. The participants stated that they did not want to make mistakes. Thus, formal leaders and colleagues should affirm nurses’ accountability for nursing activities, i.e. what they are allowed to decide and when they practise satisfactorily, otherwise they will struggle with uncertainty.

*It’s when I get the feeling*,* or someone says to me that ‘you’re doing a good job*,* you performed the task in a good way’*,* then*,* I can be accountable. In other words*,* I notice that my colleagues and the formal leader trust me or they tell me that I’m doing my job satisfactorily* (Nurse 9).

Negotiating accountability for nursing activities was also needed as colleagues did not always comprehend what the nurses’ leadership involved and which nursing activities the nurses were accountable for within the home healthcare context.

*It would help if colleagues also knew what nurses do in their daily practice of leadership. This will make our accountability for nursing activities a little easier and contribute to better acceptance of our work*,* and colleagues will know that I have a leadership role* (Nurse 8).

For colleagues to understand what is involved in nurses’ leadership in daily practice, the nurses needed to navigate daily interactions with colleagues to make their leadership more visible. The nurses often asked them to perform nursing activities, i.e. they delegated work tasks, saying what should be done, who should do it, and how. The nurses could not do all the nursing activities themselves. However, when colleagues did not quite comprehend what nurses’ leadership involved and which nursing activities the nurses were accountable for within the home healthcare context, the nurses considered delegating activities to be demanding. This was especially the case when colleagues seemed less interested or argued they could not perform the delegated nursing activities. “*When I take on leadership for getting tasks done*,* it can be a struggle*,* as I often ask myself ‘How am I going to get everything done when a colleague is not interested in doing the delegated tasks”* (Nurse 13). In such cases, the nurses negotiated accountability for nursing activities and occasionally defended their delegation. They stated that they had to defend the work they do. However, having to negotiate accountability like this could trigger feelings of uneasiness, irritation or discomfort, meaning the nurses sometimes skipped delegation and performed the nursing activities themselves. *“We are not formal leaders*,* so we cannot put ourselves forward and claim* ‘*now I’m going to take over the tasks and you do this and that’. We must be a little careful*” (Nurse 9). Here it is noteworthy that the nurses were careful when negotiating accountability for nursing activities, as they recognised that they were not formal leaders, and they could not automatically delegate nursing activities to colleagues in order to get things done. As a result, they sometimes did not delegate, even when they would have preferred to.

### Gaining an overview of nursing activities

Gaining an overview of nursing activities can be perceived as another aspect that may constitute the nurses’ leadership in daily interactions in the home healthcare context. Gaining an overview was critical as it involved focusing on current nursing activities, including new information and follow-up tasks for the day, the patient’s condition and whether any deterioration needed acting upon. This usually included a professional assessment of anticipated patient needs.

*When you have leadership in nursing you have an overview of everything and everything that is happening*,* like*,* how many patients need help*,* often it’s about twenty patients*,* and you have a responsibility for them even if you haven’t visited them in their homes. You have to be informed about their situation and what’s changed*,* or whether they have special needs to follow up […] However*,* sometimes I have an overview or take the lead more*,* and sometimes I can relax a little* (Nurse 8).

Although gaining an overview of nursing activities was closely related to a focus on professional commitment, there was no guarantee that the nurses were always fully updated. By its very nature, the home healthcare context contains a certain amount of uncertainty related to the nursing activities it encompasses and how to gain an overview of these. The nurses and their colleagues often worked independently in patients’ homes and sometimes performed nursing activities they were not familiar with, or they had to visit patients unknown to them. Nevertheless, no matter who had been on a home visit, the nurses still wanted to gain an overview of the performed nursing care. “*We have overall responsibility for the patients and make sure that they receive the treatment they should have. We must organize the day and take control of everything we’re going to do* (Nurse 5). Here the nurse stated that gaining an overview of nursing activities implied the nurses had to take some form of overall control. This control encompassed not only their own nursing activities but also those of their colleagues. Essentially, it involved ensuring that both the nurses themselves and their colleagues completed their nursing responsibilities.

Due to the nurses’ commitment to their colleagues, gaining an overview of nursing activities was crucial, as it involved helping colleagues in a way that contributed to making the working day go as smoothly as possible for everyone. While the nurses ensured that colleagues reporting to them maintained an appropriate professional nursing standard, they also had confidence in their colleagues’ capacity to be present for and help the patients. The nurses stated that they relied on their colleagues to provide them with information of relevance for nursing activities. However, there was no guarantee they would always have the detailed data necessary to assess a patient’s condition and suitable care. Some colleagues had less formal education and did not have satisfactory professional knowledge, which meant they were less insightful about their nursing activities. The nurses supported and guided these colleagues to find a solution, ensuring the best care for patients and bringing their activities to completion.

*The colleagues with less formal education often have as good an overview of patients as the nurses. We are mutually dependent on each other*,* and none of us would have managed without each other. But then it’s probably ‘a bit automatic’ that the nurses have an extra responsibility and overview*,* whether they want to or not because they are skilled* (Nurse 13).

The colleagues frequently expected the nurses to have an overall overview of the daily nursing activities, stating that this expectation was ‘a bit automatic’ (Nurse 13). Describing it this way indicates they frequently expected the nurses to have an overview of all activities within the home healthcare context and such expectations were often articulated without further negotiation. However, the nurses sometimes waited to receive relevant information from their colleagues before they could state they had gained an overview of the daily nursing activities. In fact, an overview was gained solely based on how far the nurses and colleagues interacted. Working with colleagues who followed up and completed nursing activities in a professional way was appreciated. The individual nurse gets control and thus gains an overview of the performed nursing activities. A nurse stated that “*Together we get better*,* and I achieve better control over what I do and how to complete the tasks*” (Nurse 7).

### Managing nursing activities within a limited time

Managing nursing activities within a limited time can be considered a critical aspect constituting leadership. There is limited time available within the home healthcare context to determine a course of action to work efficiently. Limited time causes the nurses to estimate how much time to spend on each nursing activity to perform activities properly. “*We only have the time we have*,* so we just have to do it as efficiently as possible*,* implying that we have done all the tasks of the day and properly done the tasks*” (Nurse 8). One course of action involved the nurses deciding where to start, or what nursing activity to do first and what to do last. Although the nurses continually had to manage nursing activities within a limited time, time management especially came into play when the working day was hectic and one or several urgent nursing activities had to be performed. To support their time management the nurses used so-called ‘patient lists’. These served as a kind of written dialogue between colleagues using the lists and provided a course of action so that the nurses could manage daily nursing activities. In a way, the patient lists created some amount of predictability for the nurses, as they included patient assignments and the time allocation for each task, e.g. how many minutes each patient visit should take. These patient lists were continually updated, along with patient assignments and time frames, which could be moved from one day to the next. It was stated that formal leaders and colleagues added nursing activities to the patient lists with messages for the nurses. These additional activities were often tasks such as taking samples for testing, checking that the blister card was dosed correctly according to the medical prescription, or inserting pharmaceuticals into medical dispensers – activities that could be time-consuming.

*Colleagues write on the patient lists if new things happen*,* whether there are medication changes from the doctor*,* and whether patients have got a new medicine cure. So*,* I have to check if there is anything new about the tasks or if the lists are correct* (Nurse 8).

However, time management of nursing activities was influenced by the fact that nursing activities often took longer than estimated and could upset the schedule. Unforeseen activities occurred, such as colleagues asking for help with their tasks. It was easy for colleagues to ask for help, but the nurses could not always help by taking over colleagues’ activities, as they had to balance these with their own activities. For this reason the nurses told their colleagues they had to figure out for themselves how to perform tasks. Alternatively, they chose to explain or show their colleagues how to perform a task.

*Now I have started to explain how colleagues should do things themselves. When colleagues say they can’t do a task*,* I tell them that they must find out themselves or I can show them straight away instead of us sitting and talking about the task* (Nurse 7).

To manage their nursing activities within a limited time and avoid time pressure, the nurses planned their driving to and between patients’ homes, as distances could be very long, ranging from five minutes to 45 min one way. “*We plan home visits so that time isn’t wasted on driving a car. We avoid driving long distances between each home visit and driving back and forth*,* but it doesn’t always work out”* (Nurse 2). However, even when the nurses planned their driving, they struggled to accomplish nursing activities within the allocated time. Half an hour could be estimated for wound care at one home visit and ten minutes for stoma care at another, but the driving time between the home visits was not factored into the schedule on the patient lists. One participant stated that *“this fact implies we have to ‘cut corners’” *(Nurse 7). The use of the metaphor *‘cut corners’* indicates that nurses may do nursing activities perfunctorily to save time. As individuals, nurses do things in different ways, particularly when they are under time pressure. To avoid time pressure the nurses often did more than one thing simultaneously, implying they did one part of an activity or fell into the temptation of postponing another activity. Nevertheless, it was crucial to manage nursing activities within a limited time, otherwise time pressure could influence the nurses’ ability to use their senses to see and hear, meaning they would not be aware of nursing activities that needed to be done and consequently, mistakes could be made.

## Discussion

This study contributes to the field of knowledge by identifying aspects that can be recognised as constituting leadership in registered nurses’ daily interactions in the home healthcare context. The three critical aspects identified are: negotiating accountability for nursing activities, gaining an overview of nursing activities, and managing nursing activities within a limited time. These aspects seem to be critical and can be recognised as constituting leadership. This is indicated by the fact that the nurses’ daily interactions with colleagues in the home healthcare context are to achieve a specific goal – that of accomplishing nursing activities to care for patients in need of help. In the view of Raelin [30], the nurses’ practice of leadership is constituted of daily interactions where colleagues are involved and where the nurses act as meaning-makers to accomplish nursing activities. Raelin states that meaning-makers are “intimately involved in their work settings” [31, p.6], implying that whatever meaning is articulated will constitute leadership, and controversially, leadership constitutes collaborative meaning-making in practice. In order to act as meaning-makers constituting leadership, thereby constituting collaborative meaning-making in practice, the nurses observe several daily interactions with colleagues connected to nursing activities. These interactions involved negotiating accountability for nursing activities, gaining an overview of nursing activities, and managing nursing activities within a limited time, all requiring the nurses to be observant. Raelin supports the view that meaning-makers “tend to be particularly observant people” [31, p.6], and adds that observing interactions incorporates “no special intrinsic powers other than his or her own awareness”. This implies that “the meaning is often there for the taking [31, p.5]. This indicates that nurses must not only be particularly observant but also have the courage to extract meaning from daily interactions to create a shared understanding with their colleagues and practice leadership.

Negotiating accountability for nursing activities thus entails the nurses in home healthcare achieving agreement among colleagues, which implies navigating daily interactions to clarify each nurse’s accountability. Research supports that nurses in home healthcare often perform various tasks that are not understood as nursing activities [17], and it may be unclear who is in charge of some activities. However, nurses in both municipality and specialist healthcare services sometimes perform tasks beyond their professional responsibility to provide patient care. While researchers agree there is a need for more clarity regarding nurses’ accountability for nursing activities, this study highlights that the practice of leadership is constituted by negotiating accountability. Negotiating often meant the nurses acted as meaning-makers, communicating what they would do in daily practice to their colleagues, as the latter did not always comprehend what the nurses’ leadership involved and which activities the nurses were accountable for. In their interactions, the nurses constructed a shared meaning regarding how accountability for nursing activities can emerge and unfold from day to day. Previous research supports the notion that engaging in meaning-making processes is salient in terms of professional accountability, which involves taking responsibility for nursing activities and observing interactions connected to nursing activities [10]. These processes are needed to demonstrate leadership in daily interactions [17, 20, 38]. Constructing shared meaning in daily interactions in the home healthcare context can delimit the risk of overlooking nurses’ practice of leadership [17]. In the worst case, such a risk can lead to a reduced understanding of accountability for leadership [25], which in turn may influence patient outcomes [10].

For the studied nurses in the home healthcare context, gaining an overview of nursing activities involves focusing on professional commitment and commitment to colleagues. The nurses detect meaning seen by patients, colleagues, and themselves. In this way, the nurses observe and have the courage to consolidate and frame their practice of leadership by having their own and colleagues’ nursing activities in place even though there is no guarantee that the nurses are always fully updated. Research confirms that nurses’ professional commitment concerns patient needs and ensures that colleagues reporting to them maintain appropriate nursing quality [16]. Lillsjöe et al. [22] agree that nurses have to gain an overview of their own nursing activities, as well as those of their colleagues. Such an overview involves insight into nursing activities as they evolve in time and space [39]. Norlyk et al. [16] confirm that although nurses have an overview of ongoing nursing activities, home healthcare presents difficulties in gaining an overview, and nurses in this context often hand nursing activities over to colleagues with less formal education. Nurses compensate for this lack of adequate professional education by giving feedback to colleagues with observations to follow up, including evaluating colleagues’ competence [15, 23].

Moreover, in the home healthcare context, managing nursing activities within a limited time frame requires that the nurses determine a proper course of action. As the time frame is limited, managing nursing activities involves a forward-looking approach that assists in anticipating upcoming challenges and prepares the nurses to work efficiently. In particular, patient lists appear to help the nurses start their working day with a forward-looking approach, allowing them to plan and anticipate how long different nursing activities may take. Raelin [31], states that the practice of leadership often requires being a meaning-maker with a forward-looking orientation in interactions. Research confirms that the limited time frame forces nurses in home healthcare to adopt a forward-looking approach and that this involves making professional judgments within a limited time [23]. This aligns with results of earlier studies from other healthcare contexts, and that indicates practising leadership does not necessarily depend on the specific context in which nursing is performed [13, 27]. Lack of time can be a barrier to the practice of leadership [13, 40], for example, when nurses have to spend time contacting other healthcare professionals, which nurses often consider unnecessary and time-consuming [18], or when they must constantly assess the urgency of nursing activities to prioritize them. This may result in the nurses tending to focus on the completion of tasks [5] rather than on their ability to practise leadership in terms of reflection, support and supervision of their peers [22].

On the other hand, it is not sufficient for the nurses to merely be observers and to have the courage necessary to detect the meaning of ‘being there for the taking’ in their daily interactions to constitute their practice of leadership in the home healthcare context. In relation to practising leadership, Raelin states that “what has been lacking is often the courage necessary to detect and then act upon it” [31, p.5]. Thus, this study highlights that to constitute the practice of leadership, the nurses have to act upon the meaning of their daily interactions connected to nursing activities. Acting upon the meaning of their daily interactions involves the nurses actually acting as meaning-makers in negotiating accountability for nursing activities and gaining an overview of nursing activities and managing these within a limited time frame. To act involves articulating meaning, which indicates that constituting leadership needs to be a subject for interpretation. In Raelin’s view [31], leaders are meaning-makers who recognise that leadership needs to be articulated in interactions and expressed in how they and others behave together in their daily interactions. Thus, it is not surprising that this study indicates that negotiating accountability, gaining an overview of nursing activities, and managing nursing activities within a limited time can never be underestimated in daily interactions related to nurses’ practice of leadership. However, these aspects need to be interpreted, implying the nurses both articulate an evolving meaning of daily interactions connected to nursing activities and adjust their practice of leadership accordingly.

### Strengths and limitations

The study’s research design was considered suitable as a strategy to ensure trustworthiness. Credibility was also ensured, as the sample consisted exclusively of eligible female nurses with experience relevant to the study’s aim. While the research approach was inductive, the study does not rely on any particular theoretical framework to perform a deductive analysis of the empirical material, implying the analysis was not conducted in a deductive way. The data collection concluded when the interviews no longer attained substantial information, and the thematic analysis approach was appropriate, as it helped to conduct the analysis systematically. The analysis has also been sufficiently described, providing transferability to the process. The theorization of this study’s results is considered useful, contributing to a re-contextualization in a broader context. However, there are some limitations to the research design of this study. The concept of leadership, as framed by Raelin [31], may not fully capture the complexities of nurses’ practice of leadership in home healthcare and limit the study’s perspective of leadership. Integrating several theoretical frameworks could therefore have been beneficial. That said, using other frameworks would not necessarily have resulted in other empirical findings, as the analysis process was data-driven. Nevertheless, aspects other than those identified in this study may constitute nurses’ leadership in home healthcare contexts, indicating the results can be seen as less critical. Furthermore, as knowledge of nurses’ practice of leadership within the home healthcare context is somewhat limited, this situation could have been an obstacle to the recruitment of eligible participants and, in turn, influenced the quality of the interview material. Nevertheless, the interview material was detailed and nuanced, and another sample would not necessarily have given more comprehensive interview data.

### Implications for practice

The results of this study have some implications for practice. An implication for organization and leadership is to support nurses in taking a position as meaning-makers to practise leadership. This involves cultivating a nursing community where nurses articulate their practice of leadership and do so to ensure the delivery of high-quality care to patients living in their homes. Leadership needs to be a subject of interpretation. One implication for nurses themselves therefore is to take a position where they act as meaning-makers who recognise their practice of leadership. In nurses’ daily interactions with colleagues, this can be done by articulating why negotiating accountability for nursing activities is necessary to reach an agreement among colleagues concerning nursing activities. Furthermore, nurses can argue why gaining an overview of nursing activities facilitates the delegation of activities to colleagues, enabling them to manage nursing activities within a limited time.

## Conclusion

This study aimed to identify what may be recognised as critical aspects constituting leadership in registered nurses’ daily interactions in three home healthcare contexts in three Norwegian municipalities. Given the study’s background, sample and methods, the nurses’ practice of leadership appears to emerge by their acting as meaning-makers in daily interactions with their colleagues. Acting as meaning-makers involves the nurses being aware of and daring to recognise aspects identified as critical in constituting the practice of leadership: negotiating accountability for nursing activities, gaining an overview of nursing activities, and managing nursing activities within a limited time. In turn, by being aware and daring to recognise what constitutes leadership, nurses have to position themselves as meaning-makers constituting leadership, and thereby as nurses who constitute collaborative meaning-making about leadership in their practice. Although this study sheds more light on nurses’ practice of leadership and what critical aspects constitute leadership in home healthcare contexts, further research is needed. The study’s results can guide further research on nurses’ practice of leadership in interactions in the home healthcare context or other contexts, such as nursing homes. One possibility is to study more closely how nurses act as meaning-makers.

## Data Availability

The datasets analysed during the current study are not publicly available for reasons of sensitivity.
